# Is qSOFA Suitable for Early Diagnosis of Sepsis Among Bacteremia Patients in Emergency Departments? Time for a Reappraisal of Sepsis-3 Criteria

**DOI:** 10.3389/fmed.2021.743822

**Published:** 2021-10-20

**Authors:** Ching-Chi Lee, Ching-Yu Ho, Po-Lin Chen, Chih-Chia Hsieh, William Yu Chung Wang, Chih-Hao Lin, Wen-Chien Ko

**Affiliations:** ^1^Clinical Medicine Research Center, National Cheng Kung University Hospital, College of Medicine, National Cheng Kung University, Tainan, Taiwan; ^2^Department of Internal Medicine, National Cheng Kung University Hospital, College of Medicine, National Cheng Kung University, Tainan, Taiwan; ^3^Department of Adult Critical Care Medicine, Tainan Sin-Lau Hospital, Tainan, Taiwan; ^4^Department of Emergency Medicine, National Cheng Kung University Hospital, College of Medicine, National Cheng Kung University, Tainan, Taiwan; ^5^Department of Management Systems, University of Waikato, Hamilton, New Zealand; ^6^Department of Medicine, College of Medicine, National Cheng Kung University, Tainan, Taiwan

**Keywords:** Sepsis-2, Sepsis-3, sepsis, bacteremia, antibiotic, source control, mortality

## Abstract

**Background:** For early recognition of patients with sepsis, quick Sequential Organ Failure Assessment (qSOFA) was proposed by Sepsis-3 criteria as initial sepsis identification outside of intensive care units. However, the new definition has subsequently led to controversy and prompted much discussion for delayed treatment efforts. We aimed to validate Sepsis-3 criteria on bacteremia patients by investigating prognostic impacts of inappropriate administration of empirical antimicrobial therapy (EAT) and delayed source control (SC) compared to Sepsis-2 criteria.

**Methods:** In the multicenter cohort of adults with community-onset bacteremia in emergency departments (EDs), adverse effects of delayed treatment efforts on 30-day mortality were examined in septic and non-septic patients by fulfilling the Sepsis-2 or Sepsis-3 criteria using the Cox regression model after adjusting independent determinants of mortality.

**Results:** Of the 3,898 total adults, septic patients accounted for 92.8% (3,619 patients) by Sepsis-2 criteria (i.e., SIRS criteria). Using Sepsis-3 criteria, 1,827 (46.9%) patients were diagnosed with early sepsis (i.e., initial qSOFA scores ≥ 2) in EDs and 2,622 (67.3%) with sepsis during hospitalization (i.e., increased SOFA scores of ≥ 2 from ED arrival). The prognostic impacts of inappropriate EAT or delayed SC (for complicated bacteremia) were both significant in septic patients with fulfilling the Sepsis-2 or Sepsis-3 (i.e., SOFA) criteria, respectively. Meanwhile, these delayed treatment efforts trivially impact prognoses of non-septic patients recognized by the Sepsis-2 or Sepsis-3 (i.e., SOFA) definitions. Notably, prognostic effects of inappropriate EAT or delayed SC were disclosed for septic patients in EDs, specifically those with qSOFA scores of ≥ 2, and prognostic impacts of delayed treatment efforts remained significant for patients initially recognized early as being non-septic (i.e., initial qSOFA scores of <2).

**Conclusions:** For patients with community-onset bacteremia, inappropriate EAT and delayed SC might result in unfavorable outcomes of patients early identified as being non-septic on ED arrival based on the qSOFA scores (by Sepsis-3 criteria). Accordingly, a more prudent diagnosis of sepsis adopted among bacteremia patients in the ED is necessary.

## Introduction

Sepsis is a worldwide public health burden that can lead to substantial mortality and cause considerable healthcare costs (1). It is evident that the early identification and treatment of sepsis can result in favorable outcomes (2, 3). However, the early identification of sepsis is difficult given its extensive variety of clinical presentations (4). Therefore, the Third International Consensus Definitions for Sepsis and Septic Shock (Sepsis-3) proposed the quick Sequential (sepsis-related) Organ Failure Assessment (qSOFA) as a replacement for the systemic inflammatory response syndrome (SIRS) criteria issued by previous Sepsis-2 criteria (5) for the prompt diagnosis of sepsis outside the intensive care units (ICUs) as SIRS scores were deemed to have unsatisfied specificity and sensitivity in detecting septic patients (6). Although the clinical applications of the revised sepsis definition have been discussed for different patient populations (7–9), there is a lack of consensus regarding its representativeness and whether or not it impedes detection and treatment efforts (10, 11).

Similar to sepsis, bacteremia is a life-threatening infection linked to high mortality and morbidity, and is generally acknowledged to be a presentation of systemic infections (12). More importantly, the prognostic advantages of appropriate administration of empirical antimicrobial therapy (EAT) and prompt source control (SC), particularly in critically ill individuals, have been emphasized in bacteremia cases (13, 14). Because the information on blood cultures (BCs) was not timely captured by the first-line clinicians, particularly for bacteremia patients initially categorized as non-septic individuals, clinicians pay less attention and this thereby results in delayed treatment efforts. Accordingly, we were concerned that inappropriate EAT and delayed SC might result in the unfavorable outcome of bacteremia patients initially categorized as non-septic according to the Sepsis-3 criteria before BC results were recognized.

For external validation of sepsis definitions on adults with community-onset bacteremia, this study aimed to investigate the adverse effects of delayed treatment efforts in terms of inappropriate EAT and delayed SC on the short-term prognoses between septic and non-septic patients, respectively, identified by the SIRS (Sepsis-2) and qSOFA/SOFA (Sepsis-3) criteria.

## Methods

### Study Design and Setting

A retrospective, multicenter cohort was established in the emergency departments (EDs) of three Taiwan hospitals during the period from Jan 2016 to Dec 2019, including a university-affiliated medical center (1,400 beds) and two teaching hospitals (380 and 460 beds). For ED patients, vital signs, mental levels, and oxygenation status were routinely assessed at ED arrival and every 4 h during the ED stay. For hospitalized patients, the same parameters were periodically recorded every 4 h in general wards and every 2 h in ICUs. The ED adults (aged ≥ 18 years) with community-onset bacteremia were eligible as the target population. The study was approved by the institutional review boards (IRBs) of each participating hospital. Clinical information was obtained and reported according to the Strengthening the Reporting of Observational Studies in Epidemiology (15).

### Selection of Participants

The patients sampled with BCs in the ED were retrieved from a computer database. Patients with community-onset bacteremia were only eligible among adults with bacterial growth. Patients with contaminated BCs, those who had the uncertain outcome or incomplete clinical data, and those diagnosed with hospital-onset bacteremia, bacteremia before ED arrival, or mycobacteremia were excluded. Only the first episode of each bacteremia patient was included if multiple bacteremia episodes.

### Measurements and Outcomes

By reviewing the electronic chart, a predetermined form was adopted to capture clinical information in terms of patient demographics, comorbid severity (McCabe classification) and types, types and antimicrobial susceptibilities of causative microorganisms, bacteremia sources, types and duration of antimicrobial administration, imaging studies, types and timing of surgical or radiological interventions, and patient outcomes. For the analysis of SIRS criteria (8), qSOFA (8), and SOFA (16) scores, all the values of vital signs, mental status, oxygenation, and laboratory data within 24 h after ED arrival were firstly captured as the initial assessment. Furthermore, to meet the Sepsis-3 criterion for organ dysfunction, an increase of ≥2 points in the SOFA score from the baseline score, the highest SOFA score within 3 days after ED arrival, was recorded.

All clinical data were jointly collected by one board-certified ED physician and another infectious-disease (ID) clinician who received training from the IRB course and were blinded to the objectives and hypotheses of the present study. The recording discrepancy was solved by the discussion between the chart reviewers in periodic meetings. The primary outcome was all-cause mortality within 30 days after ED arrival (i.e., bacteremia onset).

### Microbiological Methods

The BC bottle was incubated in a BACTEC 9240 instrument (Becton Dickinson Diagnostic Systems, Sparks, MD, USA) for 5 days, in which bacteria were identified through the matrix-assisted laser desorption ionization time-of-flight mass spectrometry (MALDI-TOF MS). To ensure the administration timing of appropriate antimicrobials in each bacteremia episode, all causative microorganisms were prospectively stored for the susceptibility test (the disk diffusion method for aerobes and the agar dilution method for anaerobes) in accordance with the contemporary standard issued by the Clinical and Laboratory Standards Institute (CLSI) (17). The tested antibiotics for Gram-negative aerobes included cefazolin, cefuroxime, cefotaxime, ceftazidime, cefepime, ertapenem, imipenem, ampicillin/sulbactam, piperacillin/tazobactam, moxifloxacin, and levofloxacin. For *Streptococcus* and *Enterococcus* spp., the tested drugs were penicillin and ampicillin, respectively. Ampicillin/sulbactam, piperacillin/tazobactam, metronidazole, and moxifloxacin were tested for anaerobes. If a patient was empirically treated with an antibiotic that was not included in the susceptibility panel originally designed, the susceptibility to the indicated agent was retrospectively examined.

### Definitions

After the exclusion of contaminated sampling, bacteremia was defined as bacterial growth in the BCs drawn from peripheral or central venipuncture. The growth of potentially contaminating microorganisms in BCs, such as *Propionibacterium* species, *Bacillus* species, *Micrococcus* species, coagulase-negative staphylococci, and Gram-positive bacilli, was considered to be the contaminated sampling (18). The onset place of the bacteremia episode within the community was regarded as community-onset bacteremia, including community and long-term healthcare-associated bacteremia (13, 19). Polymicrobial bacteremia was defined as the isolation of greater than or equal two microbial species from one bacteremia episode. A previous delineated system (McCabe classification) was assessed as the comorbid prognosis (20). As shown in Supplementary Table 1, bacteremia patients with initial SIRS criteria of ≥ 2 points were recognized as septic patients based on the Sepsis-2 criteria (5). According to the Sepsis-3 definition, bacteremia patients with initial qSOFA scores of ≥ 2 at ED arrival were identified early as septic individuals and those with organ dysfunction (i.e., an increase in SOFA scores of ≥ 2 from the baseline score within 3 days after ED arrival) during hospitalization were identified as septic patients (21).

As aforementioned (13), antimicrobial administration was appropriate if the following criteria were totally fulfilled: (i) the antibiotic was active *in vitro* against all causative pathogens isolated from one bacteremia episode according to the *2021 CLSI breakpoint* (17), (ii) the antimicrobial dosage and route were administered as the recommendations issued by the *Sanford Guide to Antimicrobial Therapy 2021* (22). As the previous definition (23), the hour gap of ≥ 24 h between the ED triage and the initiation of appropriate antimicrobials was regarded as inappropriate EAT.

The bacteremia sources were determined by established definitions of the Centers for Disease Control and Prevention (24). As indicated by the Surviving Sepsis Campaign (SSC) (25), complicated bacteremia was defined by determining whether the bacteremia source is amenable to SC, e.g., the drainage of an abscess or obstructive tract, debridement of infected necrotic tissue, removal of a potentially infected device, and definitive SC of ongoing microbial contamination. Also, as previously described (26, 27), the SC appropriateness for complicated bacteremia was determined by ID–trained physicians. The period gap of ≥ 24 h from the ED triage to appropriate SC was measured as the delayed SC.

### Statistical Analysis

The Statistical Package for the Social Science for Windows (SPSS version 23.0, Chicago, Illinois, USA) was performed for statistical analyses. Continuous variables were presented as the median and interquartile range (IQR) and compared by the *t*-test. Categorical variables were expressed as numbers and percentages and compared using the chi-square test or Fisher's exact test. Using a stepwise, backward logistic regression model, all variables of 30-day mortality with *p* < 0.1 recognized by the univariate analysis were processed to recognize the independent determinant of 30-day crude mortality. Shown by the Kaplan–Meier curve, the Cox regression model was examined to compare the adverse effect of inappropriate EAT or delayed SC on 30-day mortality of septic or non-septic patients with fulfilling varied sepsis criteria, after the respective adjustment of all the independent determinants of mortality. An E-value was tested to assess the potential effect of unmeasured confounders (28). A *p* < 0.05 was considered significant.

## Results

### Characteristics of Study Subjects

**Figure 1:**
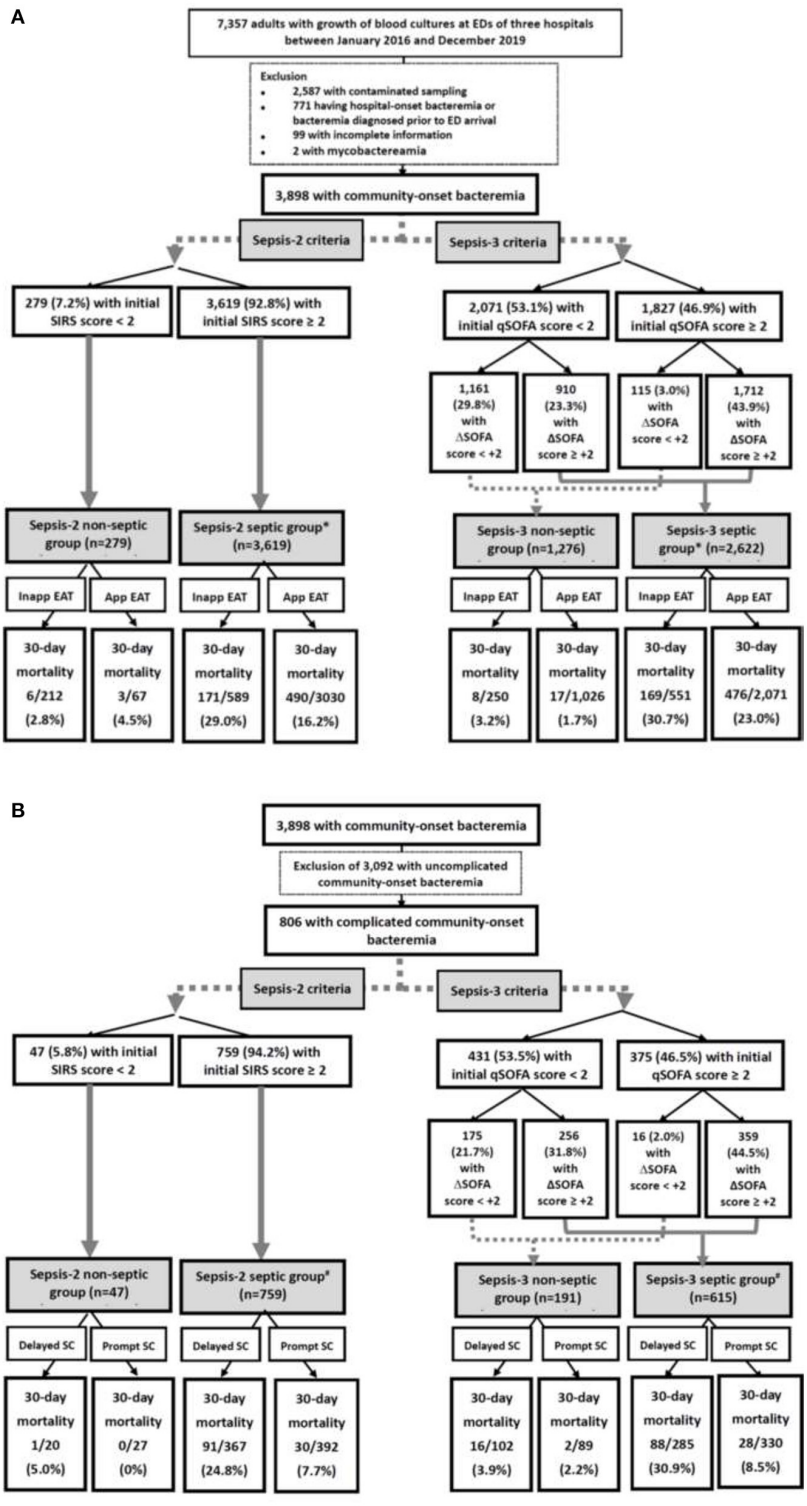
Flowchart of patient selections: **(A)** overall patients and **(B)** patients with complicated bacteremia. App, appropriate; EAT, empirical antimicrobial therapy; ED, emergency department; SIRS, systemic inflammatory response syndrome; qSOFA, quick Sequential Organ Failure Assessment; SOFA, Sequential Organ Failure Assessment; SC, source control; Inapp, inappropriate. *Indicates a significance impact of inappropriate EAT on 30-day mortality using the *Chi*-square test, compared to patients received appropriate EAT. ^#^Indicates a significance impact of delayed SC on 30-day mortality using the *Chi*-square test, compared to patients received prompt SC.

Of the total 7,357 adults with bacterial growth on BCs, 3,898 adults who had community-onset bacteremia (Figure 1A) and 806 with complicated bacteremia (Figure 1B) were eligible. Of 3,898 patients with community-onset bacteremia, their median (IQR) age was 70 (57–80) years, with male predominance (2,018 patients, 51.8%) while those received the inappropriate EAT accounted for 20.5% (801 patients). Based on Sepsis-2 criteria (i.e., SIRS criteria), septic patients accounted for 92.8% (3,619 patients). Following Sepsis-3 criteria, 1,827 (46.9%) patients were early diagnosed with sepsis (i.e., initial qSOFA scores ≥ 2) in EDs and 2,622 (67.3%) with sepsis during hospitalization (i.e., increased SOFA scores of ≥ 2 from ED arrival). The 15-day, 30-day, and in-hospital crude mortality rates were 13.6% (530 patients), 17.2% (670), and 18.2% (709), respectively. The median (IQR) of intensive care units (ICUs) and total hospitalization were 6 (3–14) and 10 (6–18) days, respectively.

Of 806 patients with complicated bacteremia, those who received delayed SC and treated with inappropriate EAT accounted for 48.0% (387 patients) and 20.7% (167), respectively. Using Sepsis-3 criteria, 375 (46.5%) patients were diagnosed with early sepsis in EDs. Septic patients accounted for 94.2% (759 patients) and 76.3% (615) as recognized by the Sepsis-2 and Sepsis-3 criteria, respectively. Because of 54 patients with septic metastasis, a total of 865 bacteremia sources were identified. The leading source of complicated bacteremia was biliary tract infections (262, 30.3%), followed by skin and soft-tissue infections (167, 19.3%), liver abscess (102, 11.8%), intra-abdominal infections (97, 11.2%), urinary tract infections (77, 8.9%), bone and joint infections (76, 8.8%), pneumonia (41, 4.7%), mycotic aneurysm (23, 2.7%), and others (17, 2.0%). The 15-day, 30-day, and in-hospital crude mortality rates were 11.8% (95 patients), 15.1% (122), and 17.2% (139), respectively. The median (IQR) of ICUs and total hospitalization were 5 (2–14) and 15 (8–28) days, respectively.

### Causative Microorganisms

Because of 390 episodes of polymicrobial bacteremia, the total 4,398 causative microorganisms were identified in overall adults. The most commonly identified microorganisms included *Escherichia coli* (1,528 isolates, 34.7%), *Klebsiella pneumoniae* (669, 15.2%), *Streptococcus* species (605, 13.8%), *Staphylococcus aureus* (511, 11.6%), anaerobes (151, 3.4%), *Pseudomonas* species (138, 3.1%), *Enterococcus* species (130, 3.0%), *Proteus* species (104, 2.4%), *Enterobacter* species (101, 2.3%), and *Salmonella* species (66, 1.5%). Methicillin-resistant *S. aureus* and ampicillin-susceptible enterococci accounted for 35.0% (179 isolates) of *S. aureus* and 89.2% (116) of enterococci, respectively. Of streptococci, 93.2% (564 isolates) were susceptible to penicillin. Overall, cefazolin, cefuroxime, ampicillin/sulbactam, moxifloxacin, cefotaxime, levofloxacin, ceftazidime, ertapenem, cefepime, piperacillin/tazobactam, or imipenem was active against 57.6, 76.3, 80.2, 80.2, 82.8, 86.2, 85.6, 90.6, 93.2, 94.9, or 100% in sequence, of Gram-negative aerobes. Ampicillin/sulbactam, moxifloxacin, piperacillin/tazobactam, or metronidazole was, respectively, active against 82.8, 92.7, 97.4, or 98.0% of the total anaerobes.

Of 884 isolates identified in 390 episodes of polymicrobial bacteremia, the leading ten were *E. coli* (194 isolates, 21.9%), *K. pneumoniae* (127, 14.4%), *Streptococcus* species (125, 14.1%), anaerobes (84, 9.5%), *Enterococcus* species (66, 7.5%), *S. aureus* (49, 5.5%), *Proteus* species (49, 5.5%), *Pseudomonas* species (36, 4.1%), *Morganella morganii* (33, 3.7%), and *Enterobacter* species (17, 1.9%). Of 390 polymicrobial bacteremia episodes, the most common source was the intra-abdominal infection (95, 24.4%), followed by pneumonia (79, 20.3%), biliary tract infections (59, 15.1%), skin and soft-tissue infections (49, 12.6%), urinary tract infections (37, %), vascular-line infections (23, 5.9%), primary bacteremia (21, 5.4%), liver abscess (8, 2.1%), bone and joint infections (7, 1.8%), and others (12, 3.1%).

### Predictors of 30-Day Mortality in Overall Patients

Using the univariate analysis, the 13 positive and 4 negative predictors of 30-day mortality were recognized in the overall cohort (Table 1). The information detailing these predictors was shown in Supplementary Result I. Through the multivariate regression model, independent determinants of 30-day crude mortality were recognized as the following (Table 1): inappropriate EAT (aOR, 1.55; *p* < 0.001), the elderly [adjusted odds ratio (aOR), 1.23; *p* = 0.046], nursing-home residents (aOR, 1.99; *p* < 0.001), bacteremia due to pneumonia (aOR, 4.17; *p* < 0.001), urinary tract infections (aOR, 0.33; *p* < 0.001), biliary tract infections (aOR, 0.41; *p* < 0.001), or liver abscesses (aOR, 0.29; *P* < 0.001), polymicrobial bacteremia (aOR, 2.00; *p* < 0.001), fatal comorbidities (aOR, 2.38; *p* < 0.001), and comorbid hemato-oncology (aOR, 1.42; *p* = 0.002), neurological diseases (aOR, 1.33; *p* = 0.02), or liver cirrhosis (aOR, 1.53; *p* = 0.002).

**Table 1:** Predictors of crude 30-day mortality in 3,898 patients with community-onset bacteremia.

**Variables**	**Patient number (%)**		**Univariate analysis**		**Multivariate analysis**	
	**Death, *n* = 670**	**Survival, *n* = 3,228**	**OR (95% CI)**	***P*-value**	**aOR (95% CI)**	***P*-value**
**Patient demographic**						
**The elderly**, **≥** **65 years**	**442 (66.0)**	**1,918 (59.4)**	**1.32 (1.11–1.58)**	**0.002**	**1.23 (1.004–1.51)**	**0.046**
Gender, male	394 (58.8)	1,624 (50.3)	1.41 (1.19–1.67)	<0.001	NS	NS
**Nursing-home resident**	**94 (14.0)**	**155 (4.8)**	**3.24 (2.47–4.24)**	** <0.001**	**1.99 (1.42–2.78)**	** <0.001**
Inappropriate EAT^*^	**177 (26.4)**	**624 (19.3)**	**1.50 (1.24–1.82)**	** <0.001**	**1.55 (1.24–1.94)**	** <0.001**
**Bacteremia source**						
**Pneumonia**	**299 (44.6)**	**349 (10.8)**	**6.65 (5.51–8.03)**	** <0.001**	**4.17 (3.30–5.26)**	** <0.001**
**Urinary tract infection**	**72 (10.7)**	**1,155 (35.8)**	**0.22 (0.17–0.28)**	** <0.001**	**0.33 (0.245–0.44)**	** <0.001**
Skin and soft-tissue infection	61 (9.1)	377 (11.7)	0.76 (0.527–1.01)	0.06	0.75 (0.54–1.05)	0.09
**Biliary tract infection**	**30 (4.5)**	**292 (9.0)**	**0.47 (0.32–0.69)**	** <0.001**	**0.41 (0.26–0.62)**	** <0.001**
**Liver abscess**	**8 (1.2)**	**130 (4.0)**	**0.29 (0.14–0.59)**	** <0.001**	**0.29 (0.13–0.62)**	**0.001**
**Polymicrobial bacteremia**	**119 (17.8)**	**271 (8.4)**	**2.36 (1.87–2.98)**	** <0.001**	**2.00 (1.52–2.62)**	** <0.001**
**Causative microorganism**						
*Escherichia coli*	167 (24.9)	1,360 (42.1)	0.46 (0.38–0.55)	<0.001	NS	NS
*Klebsiella pneumoniae*	149 (22.2)	519 (16.1)	1.49 (1.22–1.83)	<0.001	1.23 (0.96–1.58)	0.10
*Staphylococcus aureus*	121 (19.1)	389 (12.1)	1.61 (1.29–2.01)	<0.001	NS	NS
*Pseudomonas* species	45 (6.7)	93 (2.9)	2.43 (1.68–3.50)	<0.001	NS	NS
**Fatal comorbidity (McCabe classification)**	**312 (46.6)**	**716 (22.2)**	**3.06 (2.57–3.64)**	** <0.001**	**2.38 (1.88–3.00)**	** <0.001**
**Comorbidity type**						
Cardiovascular disease	344 (51.3)	1,781 (55.2)	0.86 (0.73–1.01)	0.07	NS	NS
**Hemato-oncology**	**308 (46.0)**	**903 (28.0)**	**2.19 (1.85–2.60)**	** <0.001**	**1.42 (1.13–1.78)**	**0.002**
**Neurological disease**	**217 (32.4)**	**739 (22.9)**	**1.61 (1.35–1.93)**	** <0.001**	**1.33 (1.05–1.68)**	**0.02**
**Liver cirrhosis**	**116 (17.3)**	**362 (11.2)**	**1.66 (1.32** **=** **−2.08)**	** <0.001**	**1.53 (1.17–1.99)**	**0.002**

*aOR, adjusted odds ratio; CI, confidence interval; EAT, empirical antimicrobial therapy; NS, not significant (after processing the backward multivariate regression); OR, odds ratio. Boldface indicates statistical significance with a p <0.05 under the multivariate regression model*.

* *The hour gap of ≥ 24 h between the emergency department (ED) triage and the initiation of appropriate antimicrobial therapy were regarded as inappropriate EAT*.

### Predictors of 30-Day Mortality in Patients With Complicated Bacteremia

Focusing on patients with complicated bacteremia, 12 positive and 3 negative predictors linked to 30-day mortality were identified (Table 2). The detailed data regarding these predictors were demonstrated in Supplementary Result II. In addition to inappropriate EAT (aOR, 1.67; *p* = 0.04) and delayed SC (aOR, 3.77; *p* < 0.001), five independent predictors of 30-day mortality, in terms of the elderly (aOR, 2.14; *p* = 0.002), bacteremia due to urinary tract infections (aOR, 0.29; *p* = 0.01) or liver abscesses (aOR, 0.29; *p* = 0.007), polymicrobial bacteremia (aOR, 2.28; *p* = 0.004), and fatal comorbidities (OR, 3.17; *p* < 0.001), were recognized through the multivariate regression model (Table 2).

**Table 2:** Predictors of crude 30-day mortality in 806 patients with community-onset complicated bacteremia.

**Variables**	**Patient number (%)**		**Univariate analysis**		**Multivariate analysis**	
	**Death, *n* = 122**	**Survival, *n* = 684**	**OR (95% CI)**	***P*-value**	**Adjusted OR (95% CI)**	***P*-value**
**Patient demography**						
**The elderly, 65 years**	**91 (74.6)**	**408 (59.6)**	**1.99 (1.29–3.07)**	**0.002**	**2.14 (1.33–3.45)**	**0.002**
Nursing-home resident	7 (5.7)	16 (2.3)	2.54 (1.02–6.31)	0.07	NS	NS
Inappropriate EAT^*^	**41 (33.6)**	**126 (18.4)**	**2.24 (1.47–3.42)**	** <0.001**	**1.67 (1.03–2.71)**	**0.04**
Delayed source control^**^	**92 (75.4)**	**295 (43.8)**	**4.04 (2.61–6.27)**	** <0.001**	**3.77 (2.32–6.12)**	** <0.001**
**Bacteremia source**						
Skin and soft-tissue infection	36 (29.5)	131 (19.2)	1.77 (1.15–2.73)	0.009	NS	NS
Biliary tract infection	26 (21.3)	236 (34.5)	0.51 (0.32–0.82)	0.004	0.58 (0.32–1.04)	0.07
Pneumonia	11 (9.0)	30 (4.4)	2.16 (1.05–4.44)	0.03	NS	NS
**Liver abscess**	**6 (4.9)**	**96 (14.0)**	**0.32 (0.14–0.74)**	**0.005**	**0.29 (0.12–0.71)**	**0.007**
**Urinary tract infection**	**6 (4.9)**	**71 (10.4)**	**0.45 (0.19–1.05)**	**0.06**	**0.29 (0.12–0.74)**	**0.01**
**Polymicrobial bacteremia**	**28 (23.0)**	**90 (13.2)**	**1.97 (1.22–3.17)**	**0.005**	**2.28 (1.30–4.01)**	**0.004**
**Causative microorganism**						
*Escherichia coli*	30 (24.6)	258 (37.7)	0.54 (0.35–0.84)	0.005	0.63 (0.31 −1.01)	0.05
*Staphylococcus aureus*	26 (21.3)	85 (12.4)	1.91 (1.17–3.11)	0.009	NS	NS
*Klebsiella pneumoniae*	25 (20.5)	196 (28.7)	0.64 (0.40–1.03)	0.06	NS	NS
**Fatal comorbidity (McCabe classification)**	**50 (41.0)**	**122 (17.8)**	**3.20 (2.12–4.82)**	** <0.001**	**3.17 (2.10–5.00)**	** <0.001**
**Comorbidity type**						
Cardiovascular disease	78 (63.9)	366 (53.5)	1.54 (1.03–2.30)	0.03	NS	NS
Hemato-oncology	50 (41.0)	190 (27.8)	1.81 (1.21–2.69)	0.003	NS	NS
Neurological disease	32 (26.2)	112 (16.4)	1.82 (1.16–2.85)	0.009	NS	NS
Chronic kidney disease	29 (23.8)	107 (15.6)	1.68 (1.06–2.68)	0.03	NS	NS

*aOR, adjusted odds ratio; CI, confidence interval; EAT, empirical antimicrobial therapy; NS, not significant (after processing the backward multivariate regression); OR, odds ratio. Boldface indicates statistical significance with a p <0.05 under the multivariate regression model*.

* *The time gap of ≥ 24 h between the emergency department (ED) triage and the initiation of appropriate antimicrobial therapy was regarded as inappropriate EAT*.

** *The period gap of ≥ 24 h from the ED triage to appropriate source control was measured as the delayed source control*.

### Prognostic Effects of Inappropriate EAT or Delayed SC in Septic or Non-septic Patients With Fulfilling Sepsis-2 or Sepsis-3 Criteria

**Figure 2:**
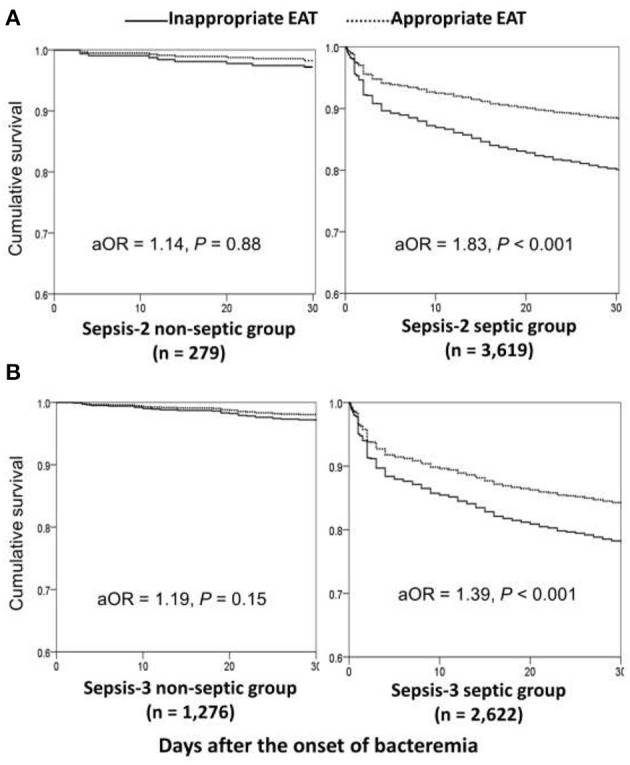
Impacts of inappropriate EAT on 30-day mortality of bacteremic patients with and without sepsis*, fulfilling the Sepsis-2 **(A)** or Sepsis-3 **(B)** criteria. aOR, adjusted odds ratio; EAT, empirical antimicrobial therapy. *After adjusting the independent predictors of 30-day mortality in overall bacteremia: the elderly, nursing-home residents, polymicrobial bacteremia, fatal comorbidities (McCabe classification), bacteremia due to pneumonia, urinary tract infections, biliary tract infections, or liver abscesses, and comorbidities of liver cirrhosis, neurological diseases, or hemato-oncology.

Using the univariate analyses in overall patients (Figure 1A), prognostic impacts of inappropriate EAT were significant in septic patients with fulfilling the Sepsis-2 or Sepsis-3 criteria compared to patients received appropriate EAT. Inappropriate EAT trivially impacted 30-day mortality in non-septic patients recognized the Sepsis-2 or Sepsis-3 criteria. Similarly, through the Cox regression model, significant prognostic impacts of inappropriate EAT were exhibited in septic patients with fulfilling the Sepsis-2 (aOR, 1.83; *p* < 0.001) or Sepsis-3 criteria (aOR, 1.39, *p* < 0*.0*01), as shown in Figure 2. Meanwhile, the prognostic impact of inappropriate EAT was independently neglected in non-septic patients identified by Sepsis-2 (aOR, 1.14; *p* = 0.88) or Sepsis-3 (aOR, 1.19; *p* = 0.15) criteria.

**Figure 3:**
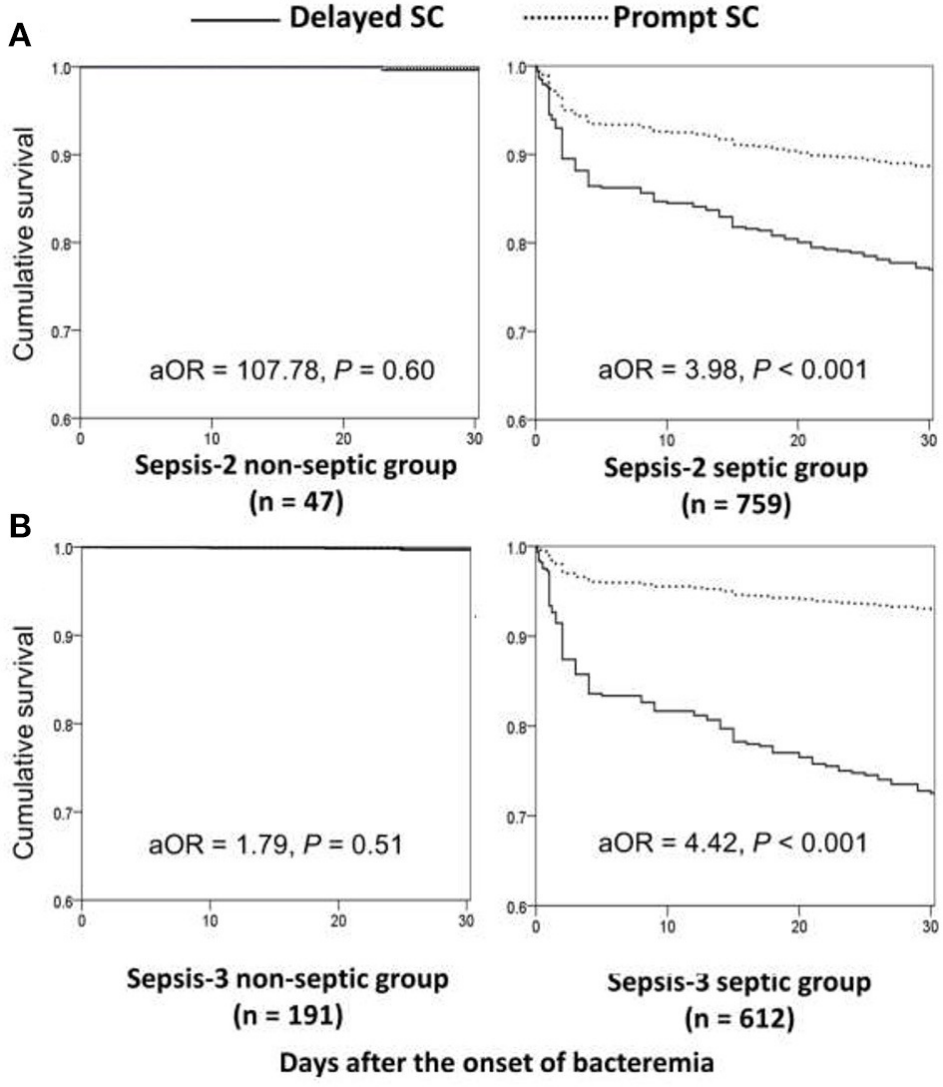
Impact of delayed SC on 30-day mortality of patients with community-onset complicated bacteremia with and without sepsis *, fulfilling the Sepsis-2 **(A)** or Sepsis-3 **(B)** criteria. aOR, adjusted odds ratio; SC, source control. *After adjusting the independent predictors of 30-day mortality in complicated bacteremia: the elderly, inappropriate empirical antimicrobial therapy, polymicrobial bacteremia, fatal comorbidities (McCabe classification), and bacteremia due to urinary tract infections or liver abscesses.

Focusing on patients experiencing complicated bacteremia (Figure 1B), significance impacts of delayed SC on 30-day mortality were observed in septic patients with fulfilling the Sepsis-2 or Sepsis-3 criteria by the univariate analyses compared to patients without delayed SC. The prognostic impacts of delayed SC remained insignificant in non-septic patients by the Sepsis-2 or Sepsis-3 criteria. Consistently, prognostic impacts of delayed SC were significant in septic patients with fulfilling the Sepsis-2 (aOR, 3.98; *p* < 0.001) or Sepsis-3 criteria (aOR, 4.42; *p* < 0.001) through the Cox regression model (Figure 3), whereas the prognostic effect of delayed SC remained neglected in non-septic individuals by the Sepsis-2 (aOR, 107.78; *p* = 0.60) or Sepsis-3 criteria (aOR, 1.79; *p* = 0.51).

### Prognostic Impacts of Inappropriate EAT or Delayed SC on Septic Patients in EDs (Recognized by qSOFA Scores)

**Figure 4:**
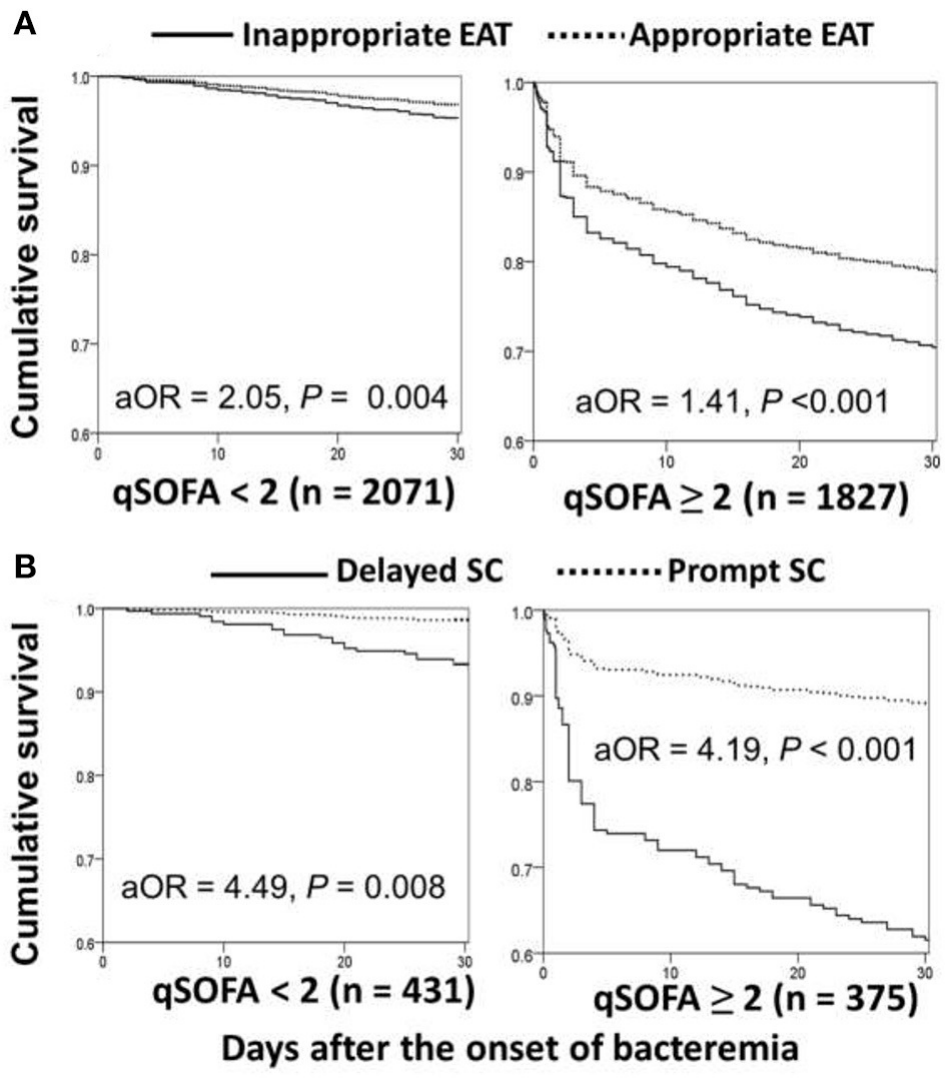
Impact of inappropriate EAT (A)* or delayed SC (B)** on 30-day mortality of patients with overall **(A)** or complicated **(B)** bacteremia, categorized into initial qSOFA of <2 and ≥ 2. aOR, adjusted odds ratio; EAT, empirical antimicrobial therapy; SC, source control. *Adjusted by the elderly, nursing-home residents, polymicrobial bacteremia, fatal comorbidities (McCabe classification), bacteremia due to pneumonia, urinary tract infections, biliary tract infections, or liver abscesses, and comorbidities of liver cirrhosis, neurological diseases, or hemato-oncology. **Adjusted by the elderly, inappropriate empirical antimicrobial therapy, polymicrobial bacteremia, fatal comorbidities (McCabe classification), and bacteremia due to urinary tract infections or liver abscesses.

Based on Sepsis-3 criteria, bacteremia patients with the initial qSOFA score of ≥ 2 in the ED were regarded as being early septic. Through the Cox regression model, prognostic impacts of inappropriate EAT were both significant in patient categorized by qSOFA scores of <2 (aOR, 2.05; *p* = 0.004) and ≥ 2 (aOR, 1.41; *p* < 0.001) (Figure 4A). Similarly, for patients with complicated bacteremia, prognostic impacts of delayed SC were both significant in patients categorized by qSOFA scores of <2 (aOR, 4.49; *p* = *0.0*08) and ≥ 2 (aOR, 4.19; *p* < 0.001) (Figure 4B).

## Discussion

Bacteremia is a life-threatening infection linked to high mortality and morbidity (12). Consistent with previous investigations, the current study demonstrated that appropriate EAT and prompt SC significantly reduce fatality of patients (13, 14, 29). In addition, the prognostic disadvantage of patients in previous studies was emphasized in our cohort. Because information detailing bacterial growth on BCs was not timely recognized by first-line physicians, based on the Sepsis-3 criteria, bacteremia patients with initial qSOFA scores of <2 were roughly captured as being non-septic in the ED. As per our concerns, adverse effects of delayed treatment efforts, in terms of inappropriate EAT and delayed SC, might result in unfavorable prognoses of such septic individuals diagnosed by initial qSOFA scores. Similar to a recent consideration that the new sepsis definition de-emphasizes intervention at earlier sepsis stages (11), our principal finding indicates that the contemporary definition is unsafe in EDs before culture information on bacteremia is recognized and, therefore, a more prudent definition of sepsis is necessary for first-line physicians.

Bacterial growth on blood cultures is the standard for the diagnoses of bacteremia and thus, this information usually delays for numerous days after culture sampling. Notably, bacteremia in the specific individual, such as the elderly (30) and cirrhotic patients (31), usually pose a diagnostic challenge for first-line clinicians due to its non-specific presentations at onset in addition to how it can be initially complicated with sepsis or, even, with sequential development of severe sepsis or septic shock (32). Accordingly, a sepsis criterion with one-size-fits-all populations to accurately recognize septic patients is crucial and essential for clinicians. Because of the annual incidence of community-onset bacteremia ranged between 43 and 154 per 100,000 in a population-based investigation (19), it is believed that the burden of community-onset bloodstream infections is comparable in magnitude to that which ED physicians face and manage daily, such as acute stroke, acute coronary syndrome, and major trauma. Therefore, this study has selected ED patients with community-onset bacteremia as the target population for validation.

Because of the considerable variations in bacteremia severity, comorbidity types and severity, immune status of patients, and distribution of causative microorganisms among patients experiencing bacteremia, the controversy regarding the prognostic impact of inappropriate EAT has been aroused. However, in the previously established investigations dealing with community-onset bacteremia (13, 33), the adverse effects of delayed treatment efforts on short-term mortality have been evidenced irrespective of whether or not patients experience the critical illness of bacteremia at onset. Patients with initial qSOFA of <2 were generally categorized as being not critically ill because of the useful performance of qSOFA in predicting short-term mortality (6). Therefore, it is reasonable that prognostic effects of delayed treatment efforts remained significant in patients regarded early as being not septic in EDs, according to Sepsis-3 criteria.

In 2016, the Sepsis-3 Task Force released a consensus statement to redefine the sepsis syndrome. It suggests early diagnosis of sepsis based on confirmed or suspected infection with qSOFA scores of ≥ 2 in the settings of out-of-hospital, emergency department, or general hospital ward, and to further recognize septic patients by an increase in SOFA scores of ≥ 2 in ICU settings (6). However, this new sepsis definition has led to controversy and prompted much discussion (10, 11). In our cohort, irrespective of whether patients assessed by the Sepsis-2 or Sepsis-3 (i.e., SOFA) criteria, inappropriate EAT and delayed SC vastly impacted the prognoses of septic patients, whereas these treatment efforts trivially affected the mortality of “non-septic” patients. Accordingly, similar to the Sepsis-2 criteria, it was evident that the application of Sepsis-3 criteria (i.e., SOFA) has remained reasonably useful for bacteremia patients. We also believed that the prompt administration of appropriate EAT and source control remained as crucial determinates of short-term mortality in bacteremia patients concurrently experiencing sepsis despite fulfilling the varied sepsis definitions. However, inferior to the Sepsis-2 criteria, our findings indicated that the early identification of sepsis by qSOFA was unsafe for such patients in the ED.

Our study has certain limitations. First, the retrospective nature of this study made it prone to recall bias during data collection. To reduce this bias, all clinical information was randomly retrieved by two physicians who were blind to the hypothesis. They also inspected medical records together to solve discrepancies. Second, regarding the effects of therapeutic strategies on patient survival, patients with uncertain mortality or incomplete clinical information were excluded. Because few proportions of patients were excluded from analyses, selection bias was negligible. Third, low E-values regarding prognostic impacts of inappropriate EAT (1.80) in overall patients and prognostic impacts of inappropriate EAT (1.91) or delayed SC (3.29) in those with complicated bacteremia were recognized herein. Thus, unmeasured confounders should be neglected. Finally, because all participant hospitals were located in southern Taiwan, our findings may be limited for generalization to other populations, which may vary in terms of causative microorganisms, bacteremia, or comorbid severity. However, the present study is the first to provide the external validation of Sepsis-3 criteria on bacteremia patients by investigating the prognostic effects of delayed treatment efforts compared to Sepsis-2 criteria.

Conclusively, for patients with community-onset bacteremia, it is evident that inappropriate EAT and delayed SC (for complicated bacteremia) impacts on short-term outcomes of the septic patients with fulfilling the Sepsis-2 or Sepsis-3 (i.e., SOFA) criteria, respectively. Meanwhile, prognostic impacts of these delayed treatment efforts were significantly neglected in non-septic patients identified by these criteria. Accordingly, despite the alteration and argument of sepsis definitions, the prompt administration of appropriate antimicrobials and source control were the crucial determinants of short-term prognoses in bacteremia patients initially experiencing sepsis syndrome. Notably, we concern the significant prognostic impacts of delayed treatment on patients early diagnosed as being not septic (i.e., initially identified by the qSOFA score of <2) outside ICUs because ED physician might pay few attention for sepsis workup and treatment for such patients, and thus, may cause the prognostic disadvantage of delayed treatment efforts could occur. Accordingly, for the safe application of Sepsis-3 criteria for bacteremia, adopting a stricter definition of sepsis in EDs is necessary.

## Data Availability Statement

The raw data supporting the conclusions of this article will be made available by the authors, without undue reservation.

## Ethics Statement

The studies involving human participants were reviewed and approved by the institutional review board of National Cheng Kung University Hospital (B-ER-109-144), Madou Sin-Lau Hospital (SLH 9919-108-006), and Tainan Sin-Lau Hospital (SLH 9919-108-009); and the requirement of obtaining informed consent was waived. Written informed consent for participation was not required for this study in accordance with the national legislation and the institutional requirements.

## Author Contributions

C-CL and W-CK conceived the study idea, designed the study, and provided expertise in infectious disease. C-CL, C-YH, C-CH, and C-HL supervised the data collection and chart reviews. C-CL provided the data of microbiological analyses and drafted this manuscript. C-CL, P-LC, and C-HL provided methodological and statistical advice on the study design and data analysis. WW and W-CK revised it carefully from a professional point of view. All authors read and approved the final manuscript.

## Funding

This study was partially supported by research grants from the Ministry of Science and Technology (MOST 109-2314-B-006-097, MOST 109-2634-F-006-023, and MOST 110-2314-B-006-068), the Ministry of Health and Welfare (MOHW109-TDU-B-211-114003), Sin-Lau Hospital (SLH-109-04), and National Cheng Kung University Hospital (NCKUH-10909031 and NCKUH-11004029), Tainan, Taiwan.

## Conflict of Interest

The authors declare that the research was conducted in the absence of any commercial or financial relationships that could be construed as a potential conflict of interest.

## Publisher's Note

All claims expressed in this article are solely those of the authors and do not necessarily represent those of their affiliated organizations, or those of the publisher, the editors and the reviewers. Any product that may be evaluated in this article, or claim that may be made by its manufacturer, is not guaranteed or endorsed by the publisher.
