# Supplementary material for: Is qSOFA Suitable for Early Diagnosis of Sepsis Among Bacteremia Patients in Emergency Departments? Time for a Reappraisal of Sepsis-3 Criteria
Source: Front Med (Lausanne). 2021 Oct 20;8:743822. doi: 10.3389/fmed.2021.743822 (PMC8563787; doi:10.3389/fmed.2021.743822)
Supplement: Supplementary file 1 [file Table_1.docx]

**SUPPLEMENTARY**

**Supplemental Table 1.** Sepsis-2 and Sepsis-3 criteria.

|  | Sepsis-2 | Sepsis-3 |
| --- | --- | --- |
| Organization | ACCP/SCCM consensus conference | SCCM/ESICM task force |
| Publication year | 1992 | 2016 |
| Definition of sepsis | The systemic inflammatory response syndrome in the presence of suspected or proven infection constitutes sepsis. | In ICU patients, sepsis is defined as life-threatening organ dysfunction caused by infections, and organ dysfunction is defined as an increase of two or more points in the SOFA score. Moreover, for adult patients outside ICUs (out-of-hospital, emergency department, or general hospital), sepsis is early recognized by the qSOFA score ≥ 2 in patients with suspected infections, which is associated with poor outcomes due to sepsis. |
| Reference No. | No. 5 | No. 6 |

ACCP = American College of Chest Physicians; ESICM = European Society of Intensive Care Medicine; qSOFA = quick Sequential Organ Failure Assessment; SOFA = Sequential Organ Failure Assessment; ICU = intensive care unit; SCCM = Society of Critical Care Medicine.
