# Supplementary material for: Is qSOFA Suitable for Early Diagnosis of Sepsis Among Bacteremia Patients in Emergency Departments? Time for a Reappraisal of Sepsis-3 Criteria
Source: Front Med (Lausanne). 2021 Oct 20;8:743822. doi: 10.3389/fmed.2021.743822 (PMC8563787; doi:10.3389/fmed.2021.743822)
Supplement: Supplementary file 2 [file Presentation_1.pdf]

Supplementary Result I

Using the univariate analysis, the following variables were positively associated with 30-day mortality in the overall cohort: the elderly, male patients, nursing-home residents**,** inappropriate EAT, bacteremic pneumonia, polymicrobial bacteremia, causative microorganisms of *K. pneumoniae, S. aureus*, or *Pseudomonas* species, fatal comorbidities (McCabe classification), and underlying hemato-oncology, neurological diseases, or liver cirrhosis. Otherwise, bacteremia caused by urinary tract infections, biliary tract infections, or liver abscesses, and *E. coli* bacteremia were factors against 30-day mortality.

Supplementary Result II

Focusing on patients with complicated bacteremia, the following variables were positively associated with 30-day mortality using the univariate analysis: the elderly, inappropriate EAT, delayed SC, polymicrobial bacteremia, S. *aureus* bacteremia, bacteremic pneumonia, bacteremia caused by skin and soft-tissue infections, fatal comorbidities (McCabe classification), and comorbidities of cardiovascular diseases, chronic kidney diseases, hemato-oncology, or neurological diseases. Otherwise, bacteremia caused by biliary tract infections or liver abscesses and *E. coli* bacteremia were factors against 30-day mortality.
